# Prenatal Exposure to Wildfire and Autism in Children

**DOI:** 10.1021/acs.est.5c08256

**Published:** 2026-01-20

**Authors:** David G. Luglio, Xin Yu, Jane C. Lin, Ting Chow, Mayra P. Martinez, Zhanghua Chen, Sandrah P. Eckel, Joel Schwartz, Frederick W. Lurmann, Nathan R. Pavlovic, Rob McConnell, Anny H. Xiang, Md Mostafijur Rahman

**Affiliations:** † Department of Environmental Health Sciences, 5783 Tulane University School of Public Health and Tropical Medicine, New Orleans, Louisiana 70118, United States; ‡ Department of Environmental Medicine, 5925 Icahn School of Medicine at Mount Sinai, New York, New York 10029, United States; § Department of Research & Evaluation, 166700 Kaiser Permanente Southern California, Pasadena, California 91101, United States; ∥ Department of Population and Public Health Sciences, Keck School of Medicine, 5116 University of Southern California, Los Angeles, California 90089, United States; ⊥ Department of Environmental Health, Harvard T. H. Chan School of Public Health, Boston, Massachusetts 02115, United States; # Department of Epidemiology, Harvard T. H. Chan School of Public Health, Boston, Massachusetts 02115, United States; ∇ Sonoma Technology, Inc., Petaluma, California 94954, United States

**Keywords:** wildfire, PM_2.5_, autism, ASD, in utero, pregnancy, neurodevelopment

## Abstract

Chronic health effects of wildfire PM_2.5_ on neurodevelopmental outcomes are largely unknown. Therefore, the effects of wildfire PM_2.5_ on autism were assessed in a southern California-based pregnancy cohort using Cox proportional hazard models. Exposure was estimated from 2006 to 2014 at maternal addresses across pregnancy and individual trimesters using three metrics: (1) mean wildfire PM_2.5_ concentration, (2) number of days of smoke exposure, and (3) number of waves of smoke exposure. Analysis was restricted to days over specific PM_2.5_ concentration thresholds (3 and 5 μg/m^3^). Nonmovers during pregnancy (75% of cohort) were assessed in sensitivity analyses. There were 3356 autism diagnoses by age 5. Autism risk was associated with increased number of wildfire-exposed days during the third trimester and was strongest for nonmovers. Nonmover hazard ratios (HR) with exposure to 1–5, 6–10, and >10 wildfire days in the third trimester (compared to none) were 1.108 (95% CI: 1.010,1.215), 1.118 (0.957,1.307), and 1.225 (1.043,1.440), respectively. HR per wildfire wave increase (>3 μg/m^3^ for 2 consecutive days) during the third trimester were 1.073 (1.009,1.140) and 1.267 (1.054,1.205) for the entire cohort and nonmovers, respectively. There was no association with the mean wildfire PM_2.5_ concentration alone. Prenatal exposure to wildfire smoke may increase risk of autism among children.

## Graphical abstract

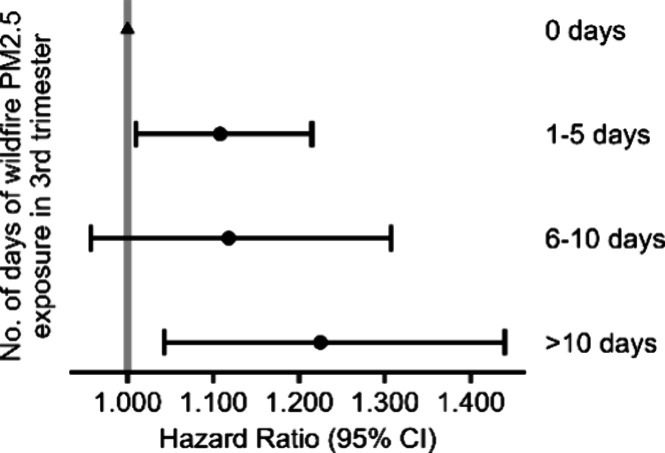

## Introduction

The threat of wildfires to the environment and health is present and increasing across the United States. More areas are experiencing burned forest and grassland, especially in the west,^1,2^ with smoke emitted from these fires spreading over large swaths of the country.^3–5^ As a result, high-impact wildfire smoke days are becoming a concern continent-wide.^6^ In fact, expected reductions in total PM_2.5_ concentrations from 2016 to 2022 as a result of regulation of man-made emissions sources of PM_2.5_ have been partially reversed as a result of wildfires, a distinct source of PM_2.5_, in many areas of the United States, including California.^7^

Autism (or autism spectrum disorder [ASD]) is a condition that is characterized by a range of divergent communicative, behavioral, and learning traits. Autistic individuals tend to have focused interests and discomfort with nonadherence to routines and may fail to recognize and understand social cues; in some cases, individuals may be nonverbal.^8,9^ Autism is caused by atypical neurodevelopment. It is known to have a major genetic contribution,^10^ but a large body of literature has shown an association between prenatal exposure to fine particulate matter (PM_2.5_) air pollution and an increased risk of autism among children.^11,12^ We have reported increased autism risk associated with prenatal exposure to several PM_2.5_ components, including elemental and organic carbons and metals.^13,14^ Autism has been associated with several PM_2.5_ sources, including tailpipe exhaust and residential heating.^15^

Wildfire PM_2.5_ has a characteristic compositional profile; it is distinguished from other PM_2.5_ sources by elevated concentrations of carbonaceous compounds, such as levoglucosan and polycyclic aromatic hydrocarbons (PAHs) like retene, as well as elements such as K, S, and some metals.^3,16–20^ Toxicity of PAHs and metals, in particular, have been well-described, including for neurological outcomes.^21–23^ PM_2.5_ emissions from different sources have been shown to have different associations with autism.^24^ A growing body of literature has demonstrated the acute effects of wildfire emitted particulate matter (PM) on several health outcomes.^3,25–27^ In particular, exposure to wildfire PM during pregnancy has been associated with decreased gestational age and birth weight,^28–32^ and neurological/cognitive effects in adults.^33,34^ While there are a growing number of studies with chronic effects of wildfire PM, there are none investigating associations with autism.^35–37^ Given the increasing impact of wildfire smoke on air quality, which may worsen with the changing climate, its impact on autism development warrants investigation.

The objective of this study was to investigate the effect of prenatal wildfire exposure on autism development in offspring, leveraging a large pregnancy cohort of the Kaiser Permanente Southern California healthcare system. Wildfire smoke PM_2.5_ concentrations were estimated by a state-of-the art validated model.^38^ We used several metrics of wildfire exposure including absolute wildfire PM_2.5_ concentration averaged over pregnancies and trimesters, number of days of wildfire events, and number of days of wildfire PM_2.5_ greater than 3 and 5 μg/m^3^, as previously suggested.^39^

## Methods

### Study Population

Participants were from a Kaiser Permanente Southern California (KPSC) pregnancy cohort, a group of mother–child pairs with singleton deliveries in system hospitals from January 1, 2006 through December 31, 2014. KPSC is an integrated healthcare system with over 4.5 million members whose membership is representative of the regional population.^40^ Maternal sociodemographic data, pregnancy health information, and addresses were extracted from KPSC’s electronic medical records (EMR) system. Addresses were geocoded by using ArcGIS.

The cohort included 245902 singleton births from 2006 to 2014 who had continued KPSC membership after age 1; among them, 41210 pairs were removed due to missing/incomplete addresses. This included participant pairs with only a street name, locality, administrative unit, or 5-digit postal code since they could not provide a certain enough location for exposure assignment. Incomplete address information leads to exposure uncertainty and possible exposure misclassification since exposures vary spatially; imprecise address information may lead to inaccurate participant location assignment which may have different exposure estimates than the true location. In addition, 84 maternal/child pairs were excluded due to the maternal age of delivery being out-of-range (i.e., <15 years or >55 years), and 234 were excluded for missing or errors in covariates (e.g., birth weight, gender, maternal race–ethnicity, etc.). The final cohort size was 204374 mother–child pairs (Supporting Information Figure S1). Characteristics of this final group are given in Table 1.

**Table 1:** Characteristics of Mother–Child Pairs, With and without Autism Diagnosis

	children, no. (%) or median (interquartile range)		
characteristics	entire cohort (*n* = 204374)	autism-diagnosed (*n* = 3356)	nondiagnosed (*n* = 201018)
sex			
male (%)	104637 (51.2)	2694 (80.3)	101943 (50.7)
female (%)	99737 (48.8)	662 (19.7)	99075 (49.3)
maternal age at delivery, median [IQR^a^], years	30.6 [26.5, 34.4]	31.5 [27.8, 35.5]	30.6 [26.5, 34.4]
parity (*N* (%))			
0	70125 (34.3)	1332 (39.7)	68793 (34.2)
1	65458 (32.0)	1060 (31.6)	64398 (32.0)
≥2	50812 (24.9)	648 (19.3)	50164 (25.0)
unknown	17979 (8.8)	316 (9.4)	17663 (8.8)
maternal education (*N* (%))			
high school or lower	64658 (31.6)	920 (27.4)	63738 (31.7)
some college	62775 (30.7)	1110 (33.1)	61665 (30.7)
college graduate or higher	74619 (36.5)	1294 (38.6)	73325 (36.5)
unknown	2322 (1.1)	32 (1.0)	2290 (1.1)
household annual income^b^ (*N* (%))			
<$30,000	10368 (5.1)	159 (4.7)	10209 (5.1)
$30,000–$49,999	57578 (28.2)	993 (29.6)	56585 (28.1)
$50,000–$69,999	64296 (31.5)	1066 (31.8)	63230 (31.5)
$70,000–$89,999	40641 (19.9)	646 (19.2)	39995 (19.9)
>$90,000	31491 (15.4)	492 (14.7)	30999 (15.4)
race/ethnicity (*N* (%))			
non-Hispanic white	50788 (24.9)	671 (20.0)	50117 (24.9)
non-Hispanic black	17465 (8.5)	300 (8.9)	17165 (8.5)
Hispanic	104085 (50.9)	1697 (50.6)	102388 (50.9)
Asian/Pacific Islander	27315 (13.4)	601 (17.9)	26714 (13.3)
other	4721 (2.3)	87 (2.6)	4634 (2.3)
any history of maternal comorbidity^c^ (*N* (%))	35237 (17.2)	674 (20.1)	34563 (17.2)
prepregnancy diabetes^d^ (*N* (%))	7731 (3.8)	190 (5.7)	7541 (3.8)
prepregnancy obesity^e^ (*N* (%))	52231 (25.6)	1029 (30.7)	51202 (25.5)
year of birth (*N* (%))			
2006–2010	98628 (48.3)	1288 (38.4)	97340 (48.4)
2011–2014	105746 (51.7)	2068 (61.6)	103678 (51.6)

a Abbreviations: IQR, interquartile range.

b Census tract level median household income.

c ≥1: diagnosis of heart, lung, kidney, or liver disease; cancer.

d Type I and Type II diabetes diagnosed before pregnancy.

e Prepregnancy BMI ≥ 30.

Children were followed from birth until the diagnosis of autism, death, loss to follow-up, or age 5, whichever occurred first. Censoring at 5 years was done to ensure that each participant had equivalent diagnosis periods. Screenings for autism diagnosis were administered at ages 18 and 24 months old during well-child visits for all children.^41^

Both KPSC (IRB No. 12075) and University of Southern California (IRB No. HS-20-00358) Institutional Review Boards approved this study with waivers of individual subject consent.

### Autism Diagnosis

The end point of this study was the diagnosis or lack of diagnosis of autism spectrum disorder through the first 5 years of life. Diagnoses were identified by ICD-9 codes 299.0, 299.1, 299.8, and 299.9 (for EMR records before October 1, 2015) and ICD-10 codes F84.0, F84.3, F84.5, F84.8, and F84.9 (after October 1, 2015) at two or more visits, as previously described.^41–44^

### Exposure Assessment

The wildfire PM_2.5_ concentration model has been described previously.^38^ This model directly outputs daily wildfire smoke PM_2.5_ concentrations on a 10 × 10 km^2^ grid across the United States and has been used in other epidemiological investigations.^5,45–48^ Gradient boosted trees were fit to estimate wildfire PM_2.5_ concentrations at all grid locations using satellite data, Hybrid Single-Particle Integrated Trajectory (HYSPLIT) model estimates, PM_2.5_ concentrations measured at the EPA Air Quality System monitors (AQS), meteorological variables (e.g., directional wind speed, mean air temperature, etc.), distance to fires, and land-use and elevation data. The cross-validated *R* ^2^ value of this model was 0.67.

Daily wildfire PM_2.5_ concentrations were assigned to the geocoded home address of each participant for the duration of each pregnancy, accounting for the change of address (as recorded in the EMR). Mean pregnancy and individual trimester concentrations were calculated for each individual and used in subsequent analysis. In addition, other measures of wildfire exposure were calculated, as previously recommended.^39^ These include the number of days of nonzero wildfire PM_2.5_ exposure and number of days of wildfire PM_2.5_ exposure > 3 and > 5 μg/m^3^ experienced by the participant at the home address. These thresholds represented the approximately 50th and 75th percentiles of wildfire exposure concentrations on wildfire days in this data set.

Additionally, daily averaged, 1 km resolution total PM_2.5_ and O_3_ concentrations were assigned to maternal addresses and averaged per individual trimester and over the entire pregnancy. Estimates were derived from an ensemble model described in detail elsewhere.^49,50^

### Covariates

Covariates included in this study were based on previously demonstrated associations and expert knowledge.^43,51–53^ Maternal-related variables included self-reported race/ethnicity, age at delivery, education, estimated household income based on census tract (per $10000), and history of comorbidity (diagnosed heart, lung, kidney, or liver disease, or cancer; yes/no). Child-related covariates included sex, birth year, and indicator variables for season of conception (i.e., dry from April to October and wet from November to March in southern California). Birth year was included as a linear variable, given the increasing trend in autism diagnoses over time. Maternal prepregnancy diabetes mellitus (yes/no) and obesity (yes/no) were also included, as they are risk factors for autism,^41^ whereas low birth weight and gestational were not included as they may be mediators in the causal pathway.^29,30,32,54–56^

### Statistical Analysis

Cox proportional hazard models estimated hazard ratios (HRs) for autism diagnosis associated with wildfire exposure metrics, adjusting for the above-mentioned covariates. For the wildfire PM_2.5_ concentrations, HRs were estimated for the entire pregnancy and each of the three trimesters in a mutually adjusted trimester model. The associations with the number of days of exposure to any wildfire PM_2.5_ and wildfire PM_2.5_ concentrations above 3 and 5 μg/m^3^ across the entire pregnancy and in the first, second, and third trimesters were evaluated. In another analysis, we created a four-level categorical variable based on the number of exposure days during pregnancy (0–5, 6–10, 11–20, and >20 days) and estimated autism risk using 0–5 days as the reference group. There were 82303 (40% of cohort), 36688 (18%), 71225 (35%), and 14158 (7%) participants in the respective categories, providing sufficient power to assess effects across these strata. Since wildfire exposure days per trimester were fewer than for the entire pregnancy, trimester-specific analyses categorized wildfire exposure as 0, 1–5, 6–10, and >10 days, with 0 days as the reference group. Similar analyses were conducted for the wildfire PM_2.5_ concentrations > 3 and > 5 μg/m^3^ with categories of 0–5, 6–10, and >10 days of exposure for both pregnancy and trimesters (reduced to three-level categorical variables to maintain sufficient power). Finally, to assess whether there was an effect of being exposed to continuous periods of wildfire, we assessed whether increased frequencies of waves of wildfire were associated with autism risk. These waves were defined over two duration periods (i.e., 2 consecutive days or more of exposure and 3 consecutive days or more of exposure) across three wildfire PM_2.5_ thresholds (i.e., >0, >3, and >5 μg/m^3^) for a total of six exposures (e.g., 2 days, >0 μg/m^3^; 2 days, >3 μg/m^3^; 3 days, >0 μg/m^3^; 3 days, >5 μg/m^3^).

Although we had access to information about residential moves during pregnancy in the EMR, we did not know the exact date and reason for the moves. Therefore, we used the midpoint between EMR-recorded address changes as the date of move and corresponding change in exposure. Fortunately, medical visits during pregnancy are frequent, but some exposure misclassification of movers is likely to have occurred. Therefore, we conducted analyses for the entire cohort and separately for mother–child pairs who did not change residential addresses during pregnancy (nonmovers), for whom exposure misclassification would have been reduced. We hypothesized that associations would be stronger among nonmovers due to reduced exposure misclassification. Summary of exposure metrics is presented in Table S1.

To address the effects of other pollutants, we adjusted the models with pregnancy or trimester averages of O_3_ concentrations or remainder PM_2.5_ concentrations. Remainder PM_2.5_ values were calculated as total PM_2.5_ minus wildfire PM_2.5_. Additionally, to adjust for potential spatial confounding effects, an indicator variable for the medical center of treatment was included as a categorical variable in the model as a sensitivity analysis.

For the analysis with absolute wildfire PM_2.5_ exposure, we scaled the HRs per pregnancy IQR increase in concentrations. For the analysis with the number of wildfire exposure days, we scaled HRs per unit day of increased exposure. Finally, for wave analysis, we scaled HRs per unit wave of increased exposure.

All statistical analyses were performed in R (version 3.5 ^57^) with packages Survival^58^ and HeatWaveR^59^ for creation of wildfire waves.

## Results

### Population Demographics

Characteristics of the study population are listed in Table 1. Mothers with autism-diagnosed children tended to be older and nulliparous and had a higher prevalence of prepregnancy diabetes and obesity. There were four times as many boys diagnosed with autism as girls. The median age of autism diagnosis in this cohort was 3.3 years. Characteristics of the nonmover (75% of total) and mover populations (25% of total) are listed in Table S2. Nonmovers were slightly older and more parous than the entire cohort and the mover population, with greater proportions of college graduates and higher prevalence of comorbidities, including prepregnancy diabetes and obesity. There were slightly higher census-tract income levels in the nonmover group.

### Exposures

About 60% of mother–child pairs were exposed to more than 5 days of wildfire smoke PM during pregnancy (Table S3). The mean exposure concentration for the cohort was 0.18 μg/m^3^ (Table 2), and the median number of days of exposure was 8, indicating that exposure was relatively infrequent. Exposure to exceptionally high concentrations of wildfire PM_2.5_ was rare (i.e., median days of exposure to wildfire PM_2.5_ concentrations > 5 μg/m^3^ was 1). Exposure levels for movers and nonmovers are presented in Table S4. These indicate that nonmovers were exposed to overall higher concentrations and greater number of days of wildfire PM_2.5_. This was consistent when averaging over the entire pregnancy, over individual trimesters, or by a wildfire event day. Exposure to wildfire waves during pregnancy and each trimester are summarized in Table S5. About 70% of participants were exposed to a 2 day wildfire PM_2.5_ event during their pregnancy.

**Table 2:** Wildfire Exposure Characteristics of Cohort over Pregnancy and Individual Trimesters

		pregnancy	1st trimester	2nd trimester	3rd trimester
wildfire PM_2.5_ concn (μg/m^3^)^a^	mean (SD^c^)	0.18 (0.21)	0.18 (0.38)	0.18 (0.36)	0.18 (0.39)
	median (IQR^d^)	0.10 (0.21)	0.04 (0.15)	0.05 (0.16)	0.05 (0.16)
no. of wildfire smoke days^b^	mean (SD)	9.55 (6.89)	3.14 (4.16)	3.42 (4.35)	2.99 (3.98)
	median (IQR)	8 (13)	1 (4)	2 (4)	1 (4)
no. of wildfire smoke days > 3 μg/m^3^ ^b^	mean (SD)	4.83 (4.65)	1.60 (2.82)	1.71 (2.92)	1.52 (2.71)
	median (IQR)	3 (7)	0 (2)	0 (2)	0 (2)
no. of wildfire smoke days > 5 μg/m^3^	mean (SD)	2.78 (3.50)	0.93 (2.09)	0.99 (2.15)	0.87 (2.00)
	median (IQR)	1 (4)	0 (1)	0 (1)	0 (1)

a Wildfire PM_2.5_ concentrations are averaged per individual across each period including non-wildfire days (where concentrations are 0 μg/m^3^).

b Note that the number of smoke days is inclusive of the number of smoke days > 3 μg/m^3^ and > 5 μg/m^3^, as number of smoke days >3 μg/m^3^ are inclusive of number of smoke days >5 μg/m^3^.

c SD, standard deviation.

d IQR, interquartile range.

### Associations with Autism

We did not find an association between the average wildfire PM_2.5_ mass concentration and autism (Figure 1a). There was an association with increased numbers of days of exposure to wildfire PM_2.5_ > 3 μg/m^3^ in the third trimester (HR [CI]: 1.014 [1.000, 1.029] per 1 day increase). Additional significant results were observed when the number of days was categorized into different levels (Table S6). Children exposed to 1–5 days of any wildfire PM_2.5_ during the third trimester had elevated risk of autism compared to those not exposed (0 days) at all (HR [CI]: 1.085 [1.002, 1.176]). The effect size was larger for wildfire PM_2.5_ > 3 μg/m^3^ (HR [CI]: 1.178 [1.018, 1.362]). Adjustment for the medical center, total PM_2.5_, and O_3_ did not appreciably change the results (Tables S7 and S8).

**Figure 1:**
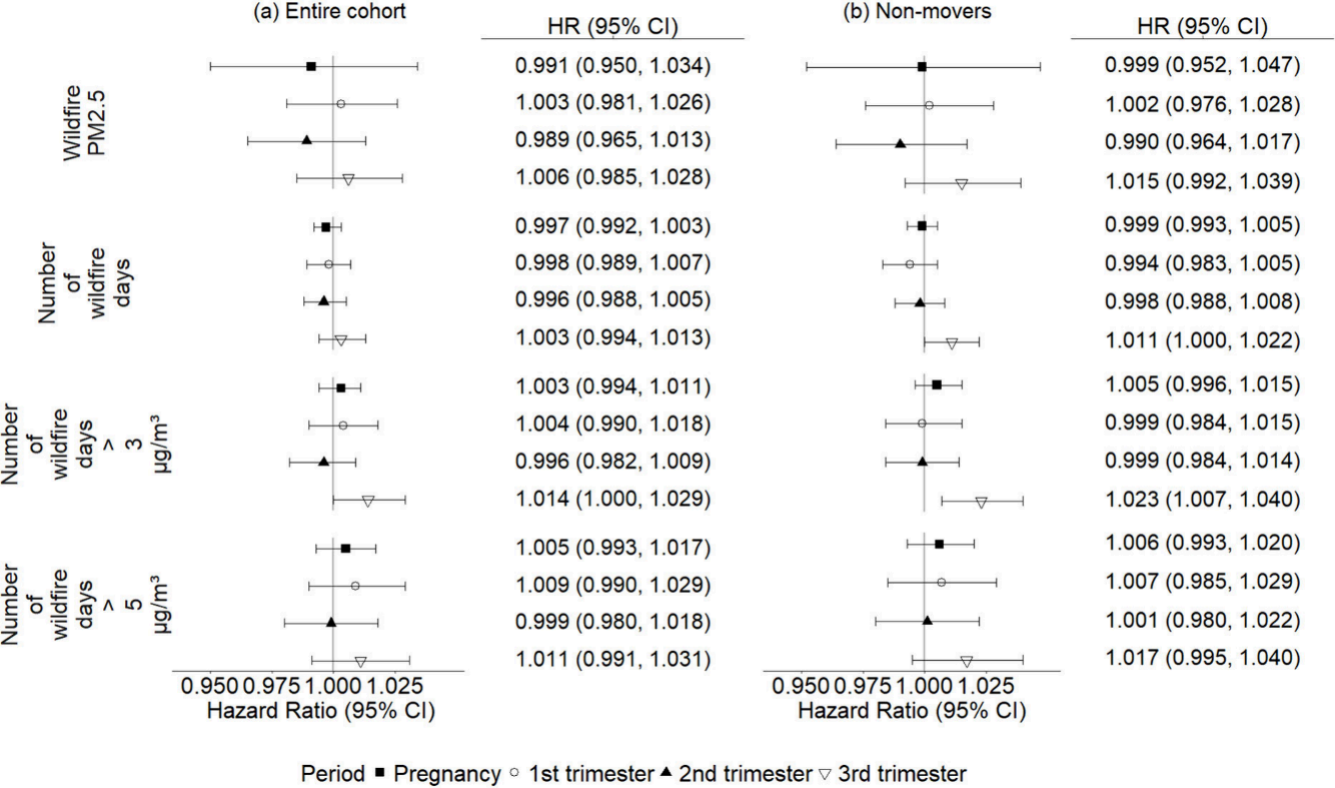
Hazard ratios between wildfire smoke exposure during entire pregnancy and each trimester and autism diagnosis in the (a) entire and (b) nonmover subsets of the cohort. PM_2.5_ concentrations are scaled per pregnancy IQR (0.21 μg/m^3^), and the number of days of exposure are scaled per day. Shapes indicate the HR for the particular period, and the bars represent the 95% confidence intervals (CIs).

When restricting the cohort to nonmovers these effects strengthened. This is apparent for increases in total days of any exposure (HR [CI]: 1.011 [1.000, 1.022]) or days with wildfire PM_2.5_ concentrations > 3 μg/m^3^ (1.023 [1.007, 1.040]) in the third trimester (Figure 1b). The results for the analyses with categorized wildfire exposure days are shown in Figure 2 and Figures S2 and S3. There was a significant increase in autism risk among mother–child pairs who were exposed to wildfire during the third trimester compared to those not exposed at all (Figure 2). The HRs of autism for exposure to 1–5, 6–10, and >10 days of wildfire, compared to none, were 1.108 (1.010, 1.215), 1.118 (0.957, 1.307), and 1.225 (1.043, 1.440), respectively. With the threshold of wildfire PM_2.5_ > 3 μg/m^3^, we also found a significant increase in autism risk associated with increased wildfire exposure days in the third trimester (Figure S2). No association was observed in the entire pregnancy, first, or second trimester analyses.

**Figure 2:**
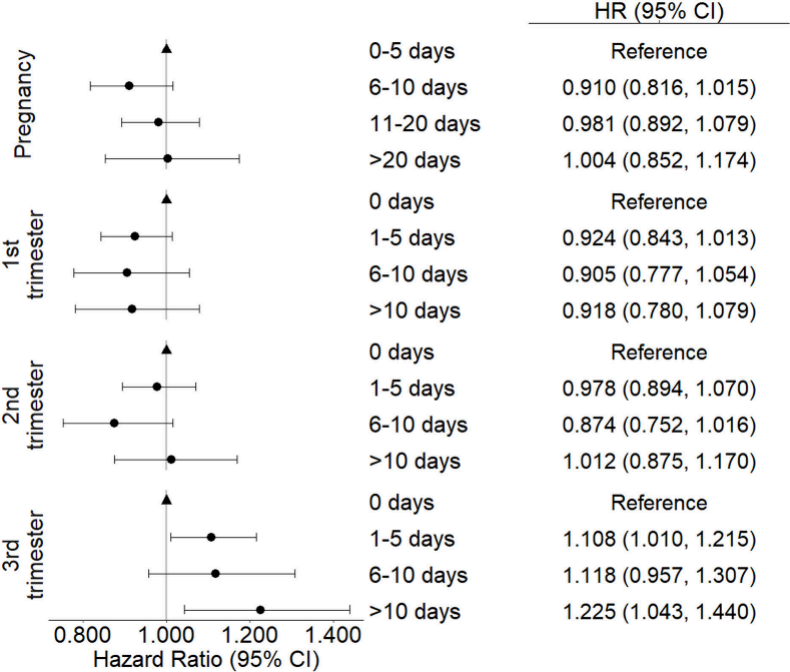
Hazard ratios for nonmovers categorized by the number of days of any wildfire PM_2.5_ exposure for each trimester. Shapes indicate the HR, with the triangle representing the reference group, and the bars represent the 95% confidence intervals (CIs).

Increases in the continuous number of days of exposure to wildfire PM_2.5_ > 5 μg/m^3^ did not show any significant associations; associations, however, were found in analysis with categorized levels of wildfire exposure days (Figure S3). Nonmover pairs exposed to over 10 days of wildfire PM_2.5_ > 5 μg/m^3^, over the entire pregnancy period, had a HR of 1.277 (1.043, 1.5621) compared with pairs exposed to five or fewer days of wildfire PM_2.5_ > 5 μg/m^3^.

The results of the analysis with wildfire exposure waves, which combined both the duration and intensity, are presented in Table 3. In the full cohort, when defining an occurrence of wildfire wave as at least two consecutive days of wildfire PM_2.5_ over 3 or 5 μg/m^3^, increased frequencies of waves during pregnancy were significantly associated with autism risk (HR [CI]: 1.045 [1.009, 1.081] and 1.057 [1.008, 1.108], respectively). Strong associations for 2 day waves were observed in the third trimester for both the entire cohort and among nonmovers. Analysis by 3 day waves yielded similar results. There were significant pregnancy-wide associations for the cohort for increases in 3 day waves of wildfire PM_2.5_ > 0 μg/m^3^ (entire cohort: 1.037 [1.003, 1.072]; nonmovers: 1.049 [1.011, 1.090]), but none for 3 day waves of wildfire PM_2.5_ > 5 μg/m^3^ across pregnancy and all trimester periods.

**Table 3:** Hazard Ratios (HRs) between Exposure to Wildfire Waves and Autism Risk in the Entire Cohort and Subset of Nonmovers

	HR (95% confidence interval)			
	pregnancy	1st trimester	2nd trimester	3rd trimester
entire cohort (*n* = 204374)				
2 days, >0 μg/m^3^	1.005 (0.983, 1.028)	1.000 (0.960, 1.042)	1.000 (0.963, 1.038)	1.029 (0.986, 1.073)
2 days, >3 μg/m^3^	1.045 (1.009, 1.081)	1.042 (0.984, 1.103)	1.027 (0.971, 1.087)	1.076 (1.012, 1.144)
2 days, >5 μg/m^3^	1.057 (1.008, 1.108)	1.068 (0.987, 1.155)	1.038 (0.959, 1.124)	1.083 (0.999, 1.174)
3 days, >0 μg/m^3^	1.037 (1.003, 1.072)	1.036 (0.975, 1.100)	1.025 (0.969, 1.085)	1.061 (0.997, 1.128)
3 days, >3 μg/m^3^	1.054 (1.002, 1.109)	1.078 (0.992, 1.172)	1.027 (0.944, 1.118)	1.077 (0.985, 1.177)
3 days, >5 μg/m^3^	1.023 (0.957, 1.094)	1.033 (0.924, 1.156)	1.009 (0.900, 1.131)	1.045 (0.926, 1.178)
nonmovers (*n* = 154036)				
2 days, >0 μg/m^3^	1.015 (0.990, 1.041)	0.985 (0.940, 1.032)	1.006 (0.964, 1.049)	1.079 (1.029, 1.131)
2 days, >3 μg/m^3^	1.068 (1.087, 1.127)	1.033 (0.968, 1.102)	1.053 (0.990, 1.122)	1.133 (1.059, 1.212)
2 days, >5 μg/m^3^	1.075 (1.020, 1.133)	1.078 (0.988, 1.177)	1.045 (0.957, 1.141)	1.127 (1.030, 1.233)
3 days, >0 μg/m^3^	1.049 (1.011, 1.090)	1.008 (0.941, 1.081)	1.038 (0.974, 1.107)	1.123 (1.049, 1.203)
3 days, >3 μg/m^3^	1.077 (1.018, 1.139)	1.071 (0.975, 1.177)	1.047 (0.953, 1.151)	1.137 (1.030, 1.254)
3 days, >5 μg/m^3^	1.050 (0.974, 1.131)	1.075 (0.948, 1.218)	1.014 (0.892, 1.152)	1.083 (0.946, 1.239)

## Discussion

This study was the first to examine the effect of prenatal exposure to wildfire on autism. The association between wildfire and autism diagnosis in offspring was assessed by utilizing a large multiethnic population-based pregnancy cohort in Southern California and a well-validated, state-of-the-art wildfire PM_2.5_ model.^38^ Increased number of days of any exposure to wildfire were associated with autism, especially in the third trimester, and the effects sizes were greater among nonmovers, although these effect sizes were small to moderate (HR up to 1.13). There was increased autism risk associated with increased frequencies of 2 day and 3 day waves of exposure across the full pregnancy and third trimester in the full cohort, and these effect estimates were stronger among nonmovers. Compared to the 2 day wave analysis, 3 day wave results show an increased effect size across the pregnancy and third trimesters for the >0 and >3 μg/m^3^ thresholds, which may be indicative of a dose–response. The lack of associations for 3 day waves of wildfire PM_2.5_ > 5 μg/m^3^, however, limits this interpretation.

A smaller sample size and reduced power among higher exposure thresholds (i.e., 5 μg/m^3^ or >10 days exposure for the categorical assignments) may explain the lack of a consistent dose–response. For example, only 8% of the cohort was exposed to 10 days or more of wildfire PM_2.5_ > 3 μg/m^3^ in the third trimester. Alternatively, more severe wildfire events, marked by higher PM_2.5_ concentrations, may result in evacuation and temporary relocation or use of indoor particle filters that could result in exposure misclassification. A previous study found that, on moderate smoke days, people stayed at home.^60^ On severe smoke days, people were more likely to temporarily relocate. Information about whether the participants temporarily relocated during a wildfire event or used indoor air filters was not available.

Estimates of effects were larger in the nonmovers than in the entire cohort. Movers likely have greater rates of exposure misclassification since dates of residential moves were interpolated between dates with different addresses. Change in address may also be linked to the presence of fires. Although we do not have information on the reason for moving, those with frequent fire exposure may choose to move. That movers were exposed to a lower average concentration and fewer days of wildfire PM_2.5_ provides some plausibility to this hypothesis. It is unclear whether other differences in characteristics between the mover and nonmover groups explained the differences between the entire cohort and nonmovers. The nonmover population was older, more parous, and more highly educated than the mover population; however, we adjusted for these covariates.

Previous studies reported associations between autism and PM_2.5_ concentrations across different pregnancy windows,^11,61^ including the first and the second and the third trimesters.^12,62,63^ In this cohort we previously found larger effects of PM2.5 (from all sources) in the first trimester.^12^ This study suggests that there is a window of susceptibility to wildfire exposure in the third trimester. Possible reasons for this different wildfire window of susceptibility are not clear. Differences in patterns of exposure and wildfire PM composition and toxicity, which are different from ambient PM from other sources, may play some role. In contrast to wildfire PM_2.5_, usual ambient PM_2.5_ concentrations are chronic at low to moderate levels; wildfire exposures are characterized by intermittent, high-concentration spikes. Increasing neuronal connectivity and organization and rapid gray matter growth are characteristic of third trimester fetal brain development^64,65^ and may be more strongly affected when there are high acute exposures. It is also plausible that there is an increased susceptibility to specific components or the compositional profile of wildfire PM during certain periods. Composition of wildfires burning at the urban interface reflects burning structures with highly toxic and poorly characterized pyrolysis products with unknown trimester-specific effects.^17^ Wildfire smoke has been reported to be enriched in K, S, Al, Si, and carbonaceous material.^3,16,18^ Carbonaceous PM components have been previously associated with autism development.^13,66^ Wildfire PM_2.5_ also generates reactive oxygen species with oxidative potential.^67,68^ Finally, wildfire smoke PM has been associated with preterm birth, and the strongest effects of increasing days of exposure were in the later pregnancy.^30^ Preterm birth, which is associated with autism, is a potential third trimester mediator of wildfire smoke exposure and autism, although other studies of preterm birth and wildfire exposure have not identified consistent trimester-specific effects.^28,29^

It is also possible that other mediators may have caused the observed associations. Wildfire events, for example, have been associated with bouts of mental health stress, including anxiety and depression;^69–72^ maternal stress during pregnancy has been associated with autism development^73^ in their children. Accordingly, these wildfire exposures may be linked to autism through stress as a mediator. Although we had adjusted for O_3_ and remainder PM_2.5_, other component or source-specific air pollution such as heavy metals could be potential confounders^13,14,24^ but were not considered. In addition, plausibly confounding temperature effects were not analyzed. Finally, other unmeasured variables, such as maternal/household activity, which could modify our findings, should be considered in future work.

No association was observed between mean wildfire PM_2.5_ concentrations and rates of autism diagnosis in this cohort across all exposure windows. These results are plausibly consistent with previous investigation examining other source-specific estimates of PM_2.5_ from a chemical transport model in this cohort; we found no association between mean biomass combustion PM_2.5_ and autism.^24^

We did not adjust for multiple testing. The wildfire exposure metrics were selected to represent different complementary features of the same underlying exposure. Notably, the statistically significant findings were concentrated in the third-trimester analyses and, more specifically, in the number of smoke-day exposures. This pattern both temporally specific (third trimester) and exposure-specific (smoke days) argues against random false positives. Moreover, the third-trimester association with smoke-day count was replicated across three analytic specifications (continuous number of smoke days, four-level categorical variable for smoke-day count, and the “wildfire wave” metric that integrates concentration and duration), which further reduces the likelihood that the result is a false discovery.

This study used a large, well-established cohort with a high retention and state-of-the-art wildfire PM_2.5_ exposure model in a region of the United States where wildfires are common and likely to increase in frequency and intensity because of climate change. Using different definitions of exposure allowed for a more robust analysis of the associations between wildfire and autism development. Some limitations include potential exposure misclassification of participants who may have evacuated during wildfire events and use of indoor air filters or personal protective equipment. Unmeasured mediators such as psychosocial stress may also have explained some of associations of exposure with autism. A variety of other potentially confounding variables, such as other source-specific or component-specific PM or other air pollutants were not included. Future work should apply multipollutant models to disentangle the independent effects of each pollutant.

Prenatal wildfire exposure was associated with autism risk in a large KPSC cohort across multiple corroborative analyses. Associations were generally strongest in the third trimester and in nonmovers, suggesting there may be a window of susceptibility during late-stage pregnancy. Further research is needed to replicate these findings, to clarify some uncertainties in the dose–response, and to identify biological pathways responsible for the associations. Public health authorities may consider prioritizing pregnant women for protection from wildfire smoke, perhaps particularly during late pregnancy.

## Supplementary Material



## Data Availability

KPSC Institutional Review Board approved this study, with waiver of informed consent with the condition that raw data remain confidential and would not be shared. Thus, due to the sensitive nature of these data, the data are not available to be shared.

## Notes

Summary of exposure metrics, additional demographic and exposure information about the cohort, and additional results for smoke day count associations and sensitivity analysis (PDF)
