# Supplementary material for: Prenatal Exposure to Wildfire and Autism in Children
Source: Environ Sci Technol. 2026 Jan 20;60(4):2907–16. doi: 10.1021/acs.est.5c08256 (PMC12874521; doi:10.1021/acs.est.5c08256)
Supplement: Supplementary file 1 [file es5c08256_si_001.pdf]

Prenatal exposure to wildfire and autism in children

David G. Luglio[1], Xin Yu[2], Jane C. Lin[3], Ting Chow[3], Mayra P. Martinez[3], Zhanghua Chen[4], Sandrah P. Eckel[4], Joel Schwartz[5,6], Frederick W. Lurmann[7], Nathan R. Pavlovic[7], Rob McConnell[4], Anny H. Xiang[3]*, Md Mostafijur Rahman[1]*

[1]Department of Environmental Health Sciences, Tulane University School of Public Health and Tropical Medicine, New Orleans, LA 70118, USA
[2]Department of Environmental Medicine, Icahn School of Medicine at Mount Sinai, New York, NY 10029, USA
[3]Department of Research & Evaluation, Kaiser Permanente Southern California, Pasadena, CA 91101, USA
[4]Department of Population and Public Health Sciences, Keck School of Medicine, University of Southern California, Los Angeles, CA 90089, USA
[5]Department of Environmental Health, Harvard T.H. Chan School of Public Health, Boston, MA 02115, USA
[6]Department of Epidemiology, Harvard T.H. Chan School of Public Health, Boston, MA 02115, USA
[7]Sonoma Technology, Inc., Petaluma, CA 94954, USA
*Md Mostafijur Rahman (mrahman8@tulane.edu) and Anny H. Xiang (anny.h.xiang@kp.org) contributed equally and serve as joint corresponding authors.

Supplementary material

Summary of exposure metrics, additional demographic and exposure information about the cohort, and additional results for smoke day count associations and sensitivity analysis

14 pages, 8 tables, and three figures

Table of contents:

Table S1 – Summary of exposure metrics	S2
Table S2 -- Characteristics of children, autistic and non-autistic (diagnosed by age of 5), of the non-mover population	S3
Table S3 – Number and proportion of cohort exposed to different numbers of smoke day exposures for entire pregnancy and each trimester	S5
Table S4 – Exposure characteristics for movers and non-movers with comparisons made by two-sample t-tests.	S6
Table S5 – Portion of cohort exposed to wildfire waves	S7
Table S6 – Hazard ratios for groups stratified based on the number of smoke days exposed across the entire pregnancy and individual trimesters.	S8
Table S7 – Sensitivity analysis of 3rd trimester models by adjustment with an indicator variable for medical center	S10
Table S8 – Sensitivity analysis of 3rd trimester models by adjustment with an indicator variable for medical center, total $PM_{2.5}$, and O_3	S11
Figure S1 -- Derivation of study sample.	S12
Figure S2 -- Hazard ratios for non-movers categorized by the number of days of wildfire $PM_{2.5}$ exposure where concentrations were > 3 μg/m^3 by exposure period.	S13
Figure S3 -- Hazard ratios for non-movers categorized by the number of days of wildfire $PM_{2.5}$ exposure where concentrations were > 5 μg/m^3 by exposure period.	S14

Table S1 – Summary of exposure metrics

Exposure	Modeling strategy
Wildfire $PM_{2.5}$ concentrations	Average across pregnancy and each trimester; modeled as a continuous variable per IQR increase
Number of days exposed to any wildfire $PM_{2.5}$	Counts during pregnancy and each trimester; modeled as<br>(1) a continuous variable per 1 day increase or (2) categorized into 4 levels: 0-5 days, 6-10 days, 11-20 days and >20 days during pregnancy or 0 days, 1-5 days, 6-10 days, and >10 days during each trimester
Number of days exposed to median level of wildfire $PM_{2.5}$ (> 3 μg/m^3)	Counts during pregnancy and each trimester; modeled as<br>(1) a continuous variable per 1 day increase or (2) categorized into 4 levels: 0-5 days, 6-10 days, 11-20 days and >20 days during pregnancy or 0 days, 1-5 days, 6-10 days, and >10 days during each trimester
Number of days exposed to high level of wildfire $PM_{2.5}$ (> 5 μg/m^3)	Counts during pregnancy and each trimester; modeled as<br>(1) a continuous variable per 1 day increase or (2) categorized into 4 levels: 0-5 days, 6-10 days, 11-20 days and >20 days during pregnancy or 0 days, 1-5 days, 6-10 days, and >10 days during each trimester
Frequency of wildfire waves represented by consecutive wildfire $PM_{2.5}$-exposed days. There are 6 versions of the definition of a wildfire wave:<br><br>At least 2- consecutive days, >0 μg/m^3;<br>At least 2- consecutive days, >3 μg/m^3;<br>At least 2- consecutive days, >5 μg/m^3;<br>At least 3- consecutive days, >0 μg/m^3;<br>At least 3- consecutive days, >3 μg/m^3:<br>At least 3- consecutive days, >5 μg/m^3	Counts of wildfire waves during pregnancy and each trimester;<br>modeled as a continuous variable per 1 count increase

Table S2 -- Characteristics of mover and non-mover children

Characteristics	Children, No. (%) or median (interquartile range)		
	Entire cohort (n = 204 374)	Non-movers (n = 154 036)	Movers (n = 50 338)
Autism diagnosis	3356 (1.6)	2563 (1.7)	793 (1.6)
Sex			
Male (%)	104 637 (51.2)	78 736 (51.1)	25 901 (51.5)
Female (%)	99 737 (48.8)	75 300 (48.9)	24 437 (48.5)
Maternal age at delivery, median [IQR[a]], years	30.6 [26.5, 34.4]	31.1 [27.1, 34.8]	29.1 [25.2, 32.9]
Parity; N (%)			
0	70 125 (34.3)	50 524 (32.8)	19 601 (38.9)
1	65 458 (32.0)	50 558 (32.8)	14 900 (29.6)
>=2	50 812 (24.9)	40 328 (26.2)	10 484 (20.8)
Unknown	17 979 (8.8)	12 626 (8.2)	5353 (10.6)
Maternal Education; N (%)			
High school or lower	64 658 (31.6)	47 637 (30.9)	17 021 (33.8)
Some college	62 775 (30.7)	46 376 (30.1)	16 399 (32.6)
College graduate or higher	74 619 (36.5)	58 185 (37.8)	16 434 (32.6)
Unknown	2322 (1.1)	1838 (1.2)	484 (1.0)
Household annual income[b]; N (%)			
<$30,000	10 368 (5.1)	7698 (5.0)	2670 (5.3)
$30,000-$49,999	57 578 (28.2)	42 925 (27.9)	14 653 (29.1)
$50,000-$69,999	64 296 (31.5)	48 316 (31.4)	15 980 (31.7)
$70,000-$89,999	40 641 (19.9)	30 860 (20.0)	9781 (19.4)
> $90,000	31 491 (15.4)	24 237 (15.7)	7254 (14.4)
Race/ethnicity; N (%)			
Non-Hispanic white	50 788 (24.9)	38 063 (24.7)	12 725 (25.3)
Non-Hispanic black	17 465 (8.5)	12 387 (8.0)	5078 (10.1)
Hispanic	104 085 (50.9)	79 004 (51.3)	25 081 (49.8)
Asian/Pacific Islander	27 315 (13.4)	21 246 (13.8)	6069 (12.1)
Other	4721 (2.3)	3336 (2.2)	1385 (2.8)
Any history of maternal comorbidity[c]; N (%)	35 237 (17.2)	27 030 (17.5)	8207 (16.3)
Pre-pregnancy diabetes[d]; N (%)	7731 (3.8)	6146 (4.0)	1585 (3.1)
Pre-pregnancy obesity[e]; N (%)	52 231 (25.6)	39 913 (25.9)	12 318 (24.5)
Year of birth, N (%)			
2006-2010	98 628 (48.3)	74 400 (48.3)	24 228 (48.1)

Characteristics	Entire cohort	Non-movers	Movers
2011-2014	105 746 (51.7)	79 636 (51.7)	26 110 (51.9)

[a] Abbreviations: IQR, interquartile range.
[b] Census tract level median household income.
[c] >=1 diagnosis of heart, lung, kidney, or liver disease; cancer.
[d] Type I and Type II diabetes diagnosed before pregnancy.
[e] Pre-pregnancy BMI>=30

Table S3 – Number and proportion of cohort exposed to different numbers of smoke day exposures for entire pregnancy and each trimester

	Number of smoke days exposed	Number of subjects	Percent of cohort
Entire Pregnancy	0-5 days[a]	82303	40%
	6-10 days	36688	18%
	11-20 days	71225	35%
	>20 days	14158	7%
1st Trimester	0 days	69430	34%
	1-5 days	93871	46%
	6-10 days	22682	11%
	>10 days	18391	9%
2nd Trimester	0 days	61982	30%
	1-5 days	98350	48%
	6-10 days	22721	11%
	>10 days	21321	10%
3rd Trimester	0 days	70942	35%
	1-5 days	95262	47%
	6-10 days	21545	11%
	>10 days	16164	8%

[a]3175 (1.6%) of mothers did not experience any smoke days at all across the entire pregnancy

Table S4 – Exposure characteristics for movers and non-movers with comparisons made by statistical tests[a].

Pregnancy		Non-movers (n = 154036)	Movers (n = 50338)	p-value
Wildfire $PM_{2.5}$ concentrations (μg/m^3)	Mean (SD)	0.183 (0.210)	0.178 (0.206)	<0.001*
	Median (IQR)	0.101 (0.209)	0.098 (0.202)	<0.001*
Number of smoke days exposed (no.)	Mean (SD)	9.57 (6.89)	9.47 (6.88)	0.007*
	Median (IQR)	8 (13)	8 (12)	0.005*
Number of wildfire smoke days > 3 μg/m^3 (no.)	Mean (SD)	4.85 (4.65)	4.77 (4.64)	0.001*
	Median (IQR)	3 (7)	3 (7)	<0.001*
Number of wildfire smoke days > 5 μg/m^3 (no.)	Mean (SD)	2.80 (3.50)	2.72 (3.47)	<0.001*
	Median (IQR)	1 (4)	1 (4)	<0.001*
Number not exposed to wildfire smoke (no.)	Count (%)	2341 (1.52)	834 (1.66)	N/A
Smoke $PM_{2.5}$ concentration during wildfire affected day (μg/m^3)	Mean (SD)	4.444 (3.119)	4.356 (3.027)	<0.001*
	Median (IQR)	3.425 (2.730)	3.382 (2.673)	<0.001*
**3rd Trimester[b]**		**Non-movers (n = 153655)**	**Movers (n = 50258)**	**p-value**
Wildfire $PM_{2.5}$ concentrations (μg/m^3)	Mean (SD)	0.186 (0.392)	0.180 (0.385)	0.002*
	Median (IQR)	0.045 (0.160)	0.046 (0.157)	0.24
Number of smoke days exposed (no.)	Mean (SD)	2.99 (3.98)	2.97 (3.98)	0.33
	Median (IQR)	1 (4)	1 (4)	0.76
Number of wildfire smoke days > 3 μg/m^3 (no.)	Mean (SD)	1.53 (2.71)	1.49 (2.68)	0.002*
	Median (IQR)	0 (2)	0 (2)	0.05
Number of wildfire smoke days > 5 μg/m^3 (no.)	Mean (SD)	0.88 (2.01)	0.84 (1.96)	0.001*
	Median (IQR)	0 (1)	0 (1)	0.12
Number not exposed to wildfire smoke (no.)	Count (%)	53568 (34.86)	17374 (34.57)	N/A
Smoke $PM_{2.5}$ concentration during wildfire affected day (SD) (μg/m^3)	Mean (SD)	4.227 (3.981)	4.150 (3.881)	0.002*
	Median (IQR)	3.010 (2.608)	2.965 (2.560)	0.003*

[a] T-tests were only performed for means comparisons. Wilcoxon tests were performed for the median comparisons.
[b] We presented data for the 3rd trimester only, as we found significant results for this trimester.
* p-value < 0.05

Table S5 – Portion of cohort exposed to wildfire waves

Exposure	Period	Number (Percent) of participants exposed to waves of wildfire PM2.5	
		**Whole cohort (n = 204 374)**	**Non-movers (n = 154 036)**
2-days, >0 μg/m^3	Pregnancy	142 401 (69.7)	107 703 (69.9)
	1st trimester	65 617 (32.1)	49 574 (32.2)
	2nd trimester	69 629 (34.1)	52 772 (34.3)
	3rd trimester	64 141 (31.5)	48 481 (31.6)
2-days, >3 μg/m^3	Pregnancy	97 124 (47.5)	73 653 (47.8)
	1st trimester	38 118 (18.6)	28 914 (18.8)
	2nd trimester	40 780 (20.0)	30 965 (20.1)
	3rd trimester	37 939 (18.6)	28 785 (18.7)
2-days, >5 μg/m^3	Pregnancy	68 044 (33.3)	51 595 (33.5)
	1st trimester	26 400 (12.9)	19 981 (13.0)
	2nd trimester	27 889 (13.6)	21 186 (13.8)
	3rd trimester	25 208 (12.4)	19 140 (12.5)
3-days, >0 μg/m^3	Pregnancy	100 483 (49.2)	76 034 (49.4)
	1st trimester	41 765 (20.4)	31 558 (20.5)
	2nd trimester	43 557 (21.3)	33 022 (21.4)
	3rd trimester	38 461 (18.9)	29 168 (19.0)
3-days, >3 μg/m^3	Pregnancy	66 465 (32.5)	50 498 (32.8)
	1st trimester	26 202 (12.8)	19 887 (12.9)
	2nd trimester	27 311 (13.4)	20 744 (13.5)
	3rd trimester	23 877 (11.7)	18 205 (11.8)
3-days, >5 μg/m^3	Pregnancy	49 198 (24.1)	37 429 (24.3)
	1st trimester	18 805 (9.2)	14 278 (9.3)
	2nd trimester	19 721 (9.6)	15 017 (9.7)
	3rd trimester	17 050 (8.4)	12 994 (8.5)

Table S6 – Hazard ratios for groups stratified based on the number of smoke days exposed across the entire pregnancy and individual trimesters.

	Period	Group	HR	95% CI
**Days > 0 μg/m^3**	Pregnancy	0-5 days	1.000	Reference
		6-10 days	0.885	(0.804, 0.974)
		11-20 days	0.964	(0.887, 1.047)
		>20 days	0.967	(0.839, 1.113)
	1st trimester	0 days	1.000	Reference
		1-5 days	0.945	(0.872, 1.024)
		6-10 days	0.938	(0.820, 1.073)
		>10 days	0.976	(0.847, 1.125)
	2nd trimester	0 days	1.000	Reference
		1-5 days	0.971	(0.897, 1.050)
		6-10 days	0.920	(0.808, 1.047)
		>10 days	0.972	(0.855, 1.105)
	3rd trimester	0 days	1.000	Reference
		1-5 days	1.085	(1.002, 1.176)
		6-10 days	1.018	(0.886, 1.169)
		>10 days	1.090	(0.942, 1.261)
	**Period**	**Group**	**HR**	**95% CI**
**Days > 3 μg/m^3**	Pregnancy	0-5 days	1.000	Reference
		6-10 days	0.956	(0.867, 1.053)
		>10 days	1.072	(0.958, 1.198)
	1st trimester	0-5 days	1.000	Reference
		6-10 days	0.968	(0.835, 1.122)
		>10 days	1.051	(0.830, 1.331)
	2nd trimester	0-5 days	1.000	Reference
		6-10 days	1.000	(0.871, 1.148)
		>10 days	0.860	(0.673, 1.099)
	3rd trimester	0-5 days	1.000	Reference
		6-10 days	1.178	(1.018, 1.362)
		>10 days	0.999	(0.765, 1.306)
	**Period**	**Group**	**HR**	**95% CI**
**Days > 5 μg/m^3**	Pregnancy	0-5 days	1.000	Reference
		6-10 days	1.001	(0.896, 1.119)
		>10 days	1.256	(0.934, 1.357)
	1st trimester	0-5 days	1.000	Reference
		6-10 days	1.032	(0.874, 1.218)
		>10 days	1.416	(0.876, 2.288)
	2nd trimester	0-5 days	1.000	Reference
		6-10 days	1.058	(0.904, 1.238)
		>10 days	0.944	(0.521, 1.710)
	3rd trimester	0-5 days	1.000	Reference

Group	HR	95% CI
6-10 days	1.046	(0.876, 1.250)
>10 days	0.994	(0.532, 1.857)

Table S7 – Sensitivity analysis of 3rd trimester models by adjustment with an indicator variable for medical center

		HR (95% CI)	
**Exposure**		**Base model**	**+ medical center**
Wildfire $PM_{2.5}$ concentration	Entire cohort	1.007 (0.986, 1.029)	1.009 (0.988, 1.031)
	Non-movers	1.016 (0.993, 1.040)	1.018 (0.994, 1.042)
Number of smoke days	Entire cohort	1.003 (0.994, 1.013)	1.005 (0.995, 1.015)
	Non-movers	1.011 (1.000, 1.022)	1.013 (1.002, 1.024)
Number of wildfire smoke days > 3 μg/m^3	Entire cohort	1.015 (1.000, 1.029)	1.014 (0.999, 1.028)
	Non-movers	1.024 (1.008, 1.040)	1.022 (1.006, 1.039)
Number of wildfire smoke days > 5 μg/m^3	Entire cohort	1.011 (0.992, 1.031)	1.012 (0.993, 1.032)
	Non-movers	1.017 (0.995, 1.040)	1.018 (0.995, 1.040)

Table S8 – Sensitivity analysis of 3rd trimester models by adjustment with an indicator variable for medical center, total $PM_{2.5}$, and O_3

Exposure		HR (95% CI)		
		Base model	+ remainder $PM_{2.5}$	+ O_3
Wildfire $PM_{2.5}$ concentration	Entire cohort	1.007 (0.986, 1.029)	1.010 (0.989, 1.032)	1.014 (0.993, 1.037)
	Non-movers	1.016 (0.993, 1.040)	1.019 (0.995, 1.043)	1.023 (0.999, 1.048)
Number of smoke days	Entire cohort	1.003 (0.994, 1.013)	1.003 (0.994, 1.013)	1.008 (0.998, 1.018)
	Non-movers	1.011 (1.000, 1.022)	1.011 (1.000, 1.022)	1.015 (1.004, 1.027)
Number of wildfire smoke days > 3 μg/m^3	Entire cohort	1.015 (1.000, 1.029)	1.016 (1.002, 1.030)	1.018 (1.004, 1.033)
	Non-movers	1.024 (1.008, 1.040)	1.024 (1.008, 1.040)	1.026 (1.010, 1.043)
Number of wildfire smoke days > 5 μg/m^3	Entire cohort	1.011 (0.992, 1.031)	1.014 (0.995, 1.034)	1.013 (0.994, 1.033)
	Non-movers	1.017 (0.995, 1.040)	1.020 (0.997, 1.043)	1.018 (0.996, 1.041)

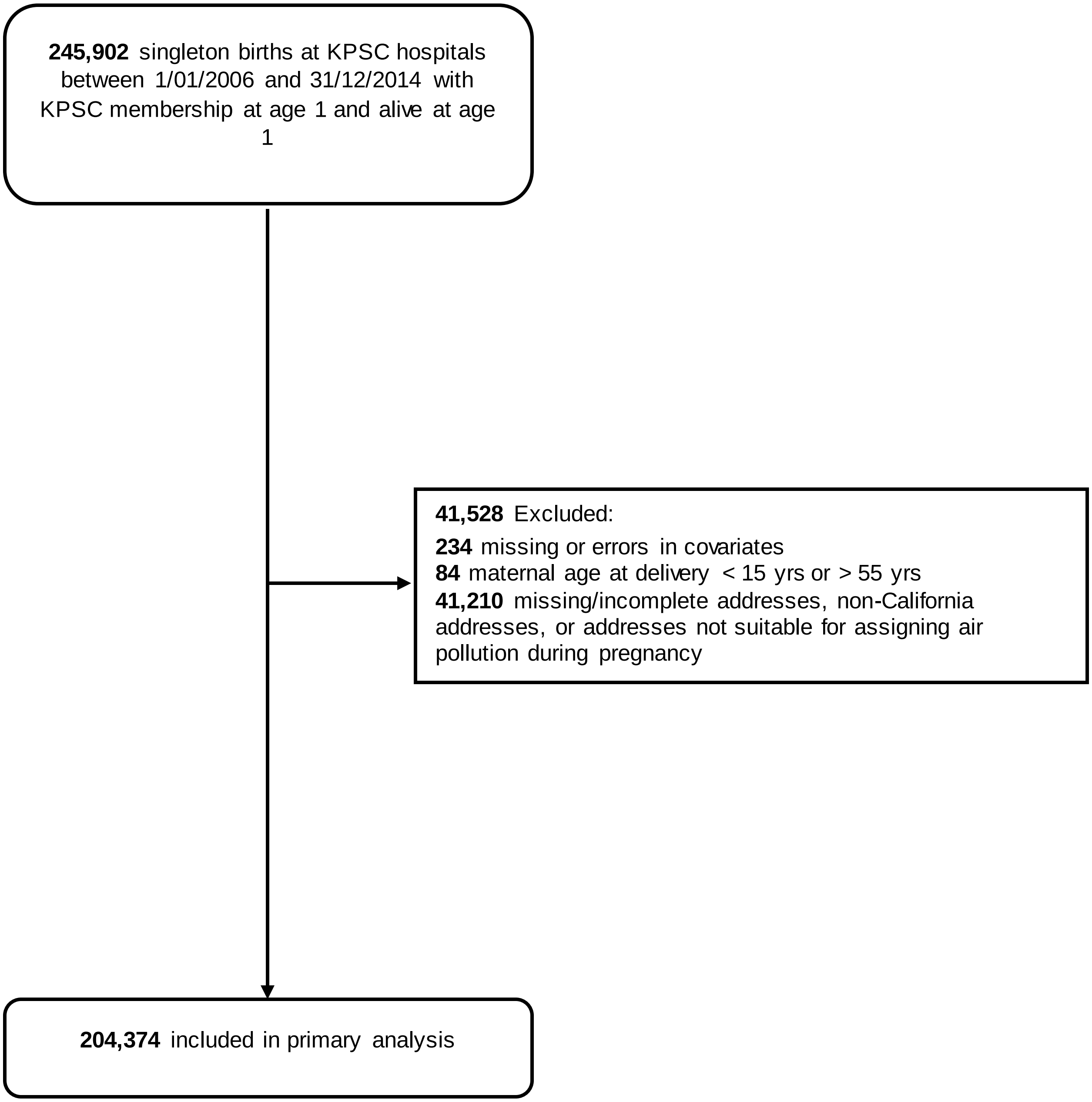

Figure S1 -- Derivation of study sample.

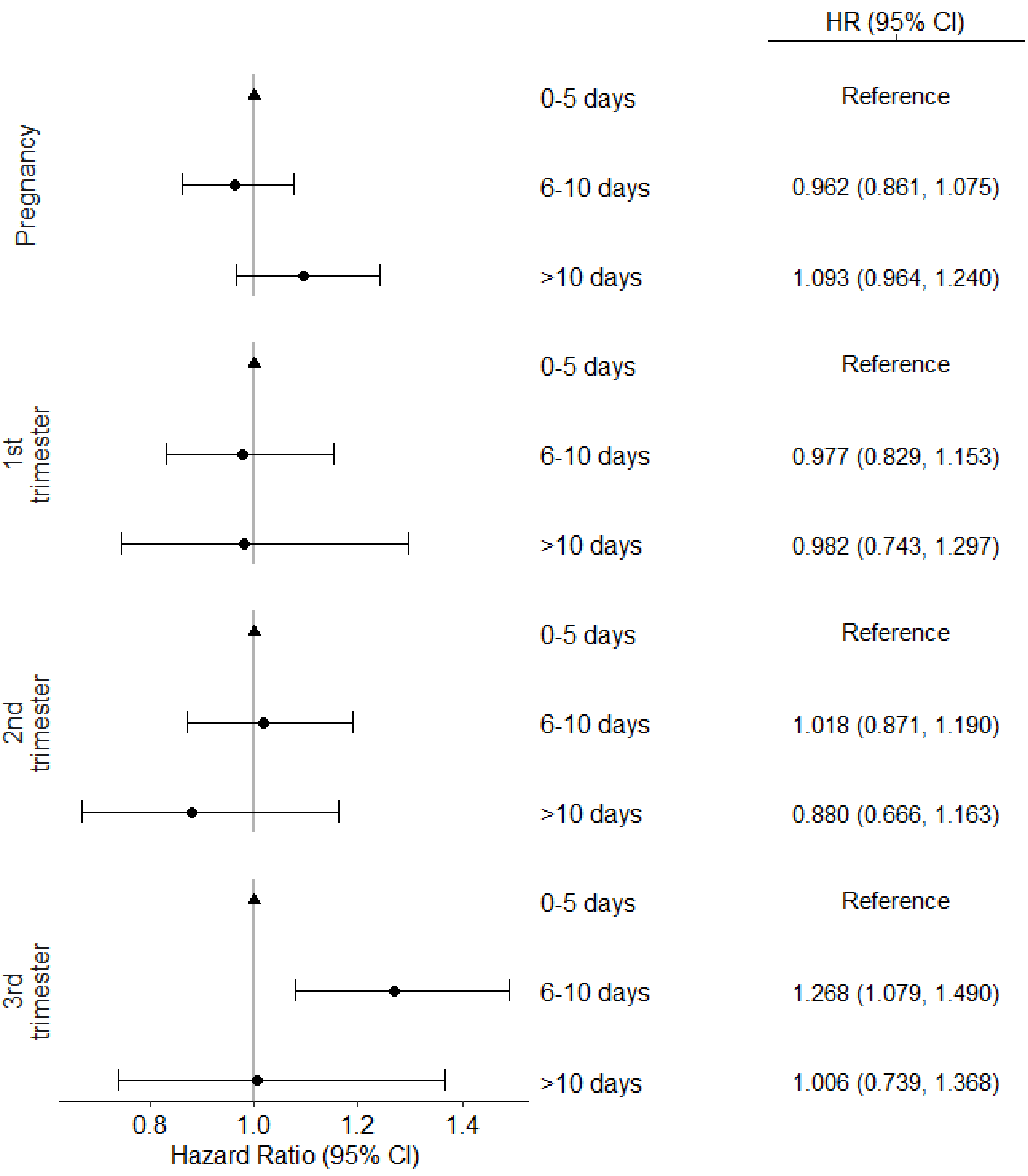

Figure S2 -- Hazard ratios for non-movers categorized by the number of days of wildfire $PM_{2.5}$ exposure where concentrations were > 3 μg/m^3 by exposure period.

Shapes indicate the HR, w ith the triangle representing the reference group, and the bars represent the 95% confidence intervals (CI).

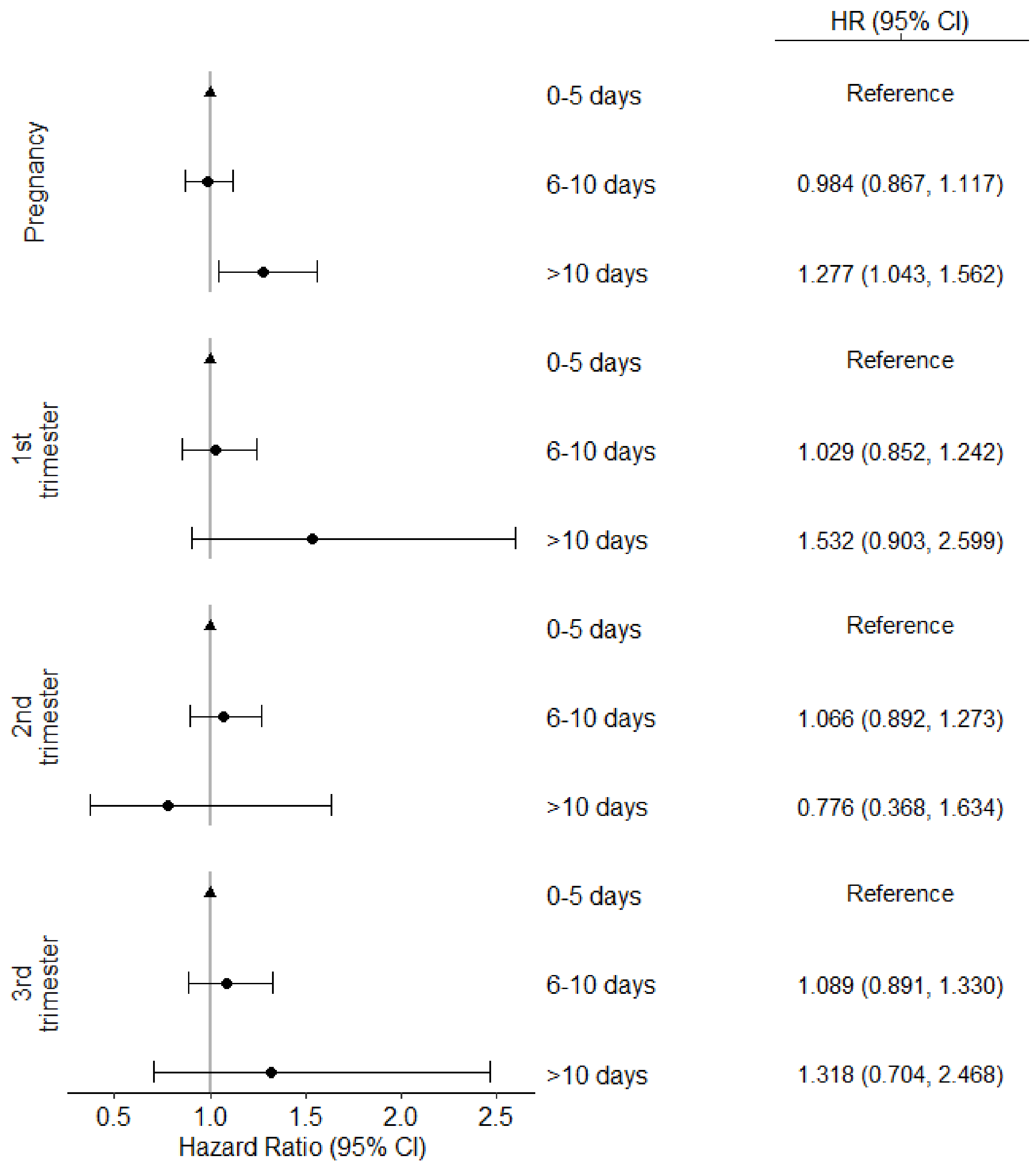

Figure S3 -- Hazard ratios for non-movers categorized by the number of days of wildfire $PM_{2.5}$ exposure where concentrations were > 5 μg/m^3 by exposure period.

Shapes indicate the HR, with the triangle representing the reference group, and the bars represent the 95% confidence intervals (CI).
